# Dental Service Utilization and Barriers to Dental Care for Individuals with Autism Spectrum Disorder in Jordan: A Case-Control Study

**DOI:** 10.1155/2020/3035463

**Published:** 2020-08-03

**Authors:** Sabha Mahmoud Alshatrat, Isra Abdelkarim Al-Bakri, Wael Mousa Al-Omari

**Affiliations:** ^1^Department of Applied Dental Sciences, College of Applied Medical Sciences, Jordan University of Science and Technology, Irbid, Jordan; ^2^Department of Prosthodontics, Faculty of Dentistry, Jordan University of Science and Technology, Irbid, Jordan; ^3^Restorative and Prosthetic Dental Sciences Department, College of Dentistry, King Saud Bin Abdulaziz University for Health Sciences, Riyadh, Saudi Arabia

## Abstract

Individuals with disabilities are at higher risk for oral diseases such as caries and periodontal disease. Therefore, regular dental care is essential to maintain oral health. However, individuals with disabilities encounter difficulties in accessing dental care. The challenges and barriers to oral care faced by individuals with autism spectrum disorder (ASD) have not been addressed in Jordan. The aim of this study was to examine the use of dental services in individuals with ASD in Jordan and identify barriers that affect their access to dental care in comparison with individuals without ASD. A case-control study was carried out among 296 parents/caregivers of individuals with ASD and individuals without ASD, which involved completion of a self-designed questionnaire. The majority of the participants in both groups had visited the dentist in the year preceding completion of the questionnaire. The main reason for visiting dental services was toothache (43%), and the least common reason was routine checkup (11.6%), with a significant difference (*P* < 0.05) observed between the two groups. Barriers including embarrassment (43.5%), a lack of specialized dental staff (28.6%), a lack of knowledge of how to treat people with disabilities (26.6%), and inadequate facilities (34%) were significantly (*P* < 0.05) more likely to be reported by individuals with ASD than the controls. In conclusion, knowing and understanding the barriers to accessing dental care could improve overall health for individuals with ASD. Furthermore, recognizing the challenges in accessing dental care for this population could help oral health professionals to minimize these difficulties.

## 1. Introduction

Disability has been defined as any condition (body or mind) with impairments, activity limitations, and participation restrictions [1]. It has been estimated that more than one billion, (approximately 15%) of the world's population, have disabilities [2]. Moderate and severe disabilities are estimated to affect 200 million people in developing countries, and the number of individuals with a disability in Jordan is approximately 250,000 [3]. Autism is one example of a disability. The American Psychiatric Association defines autism spectrum disorder (ASD) as a neurodevelopmental disorder characterized by social and communication impairment, restricted interests, and repetitive behaviors [4]. In 2013, the Diagnostic and Statistical Manual of Mental Disorders (DSM) defined ASD as a single disorder after many subtypes of ASD were merged under one definition. The term ASD incorporates autistic disorder, pervasive developmental disorder—not otherwise specified, and Asperger syndrome [5]. The prevalence of ASD is rising globally. According to the Centers for Disease Control and Prevention (CDC), one in every 59 children is diagnosed with ASD [6]. In 2018, there were 8,000 estimated cases of ASD in Jordan [7]. However, due to little research and information available on ASD, tracking the prevalence of ASD in Jordan is challenging.

Dental care is a major unmet health care need in children with special health care needs and disabilities [8, 9]. Children with ASD have greater dental needs compared to individuals without ASD [10], and in general, individuals with disabilities are more likely to have worse oral health than individuals without such disabilities [11–13]. Research has shown that individuals with ASD are more likely to have oral health problems than those without ASD. A systemic review and meta-analysis found that children and young adults with ASD have a high prevalence of dental caries and periodontal disease [11]. Furthermore, delayed tooth eruption, mouth trauma and injury (biting lips and picking at gingiva), bruxism, nonnutritive chewing (eating objects), and tongue thrusting are all oral health problems associated with ASD. The increased risk of poorer oral health in individuals with ASD might be due to specific medications, certain behaviors, and difficulty in maintaining daily hygiene [13]. For example, hyperactivity, easy frustration, unpredictable body movements, and self-injurious behavior can make regular dental care very challenging for individuals with ASD [14].

In addition to the above mentioned factors, individuals with ASD might face further challenges and barriers which could limit their access to dental care, such as low family income and a history of uncooperative behavior in a dental office [15]. Furthermore, the inability to find a dentist who has the necessary skills or willingness to work with this population groups are reasons why individuals with ASD may not make routine dental visits [15]. Dental care is essential to maintain optimal oral health. To date, there have been no studies conducted in Jordan addressing the challenges and barriers faced by individuals with ASD and their caregivers in providing the required oral care for their children.

Knowing and understanding these barriers might improve the overall health and quality of life of individuals with ASD. In addition, identifying and determining the challenges of accessing dental care for this population might help oral health professionals minimize these obstacles.

Therefore, the objectives of this study were to analyze dental service utilization and identify perceived barriers to access dental care among Jordanian individuals with ASD compared to individuals without ASD.

## 2. Materials and Methods

A cross-sectional study was conducted in individuals with ASD. A self-designed questionnaire was developed in the Arabic language, the official language of Jordan. The content and face validity of the self-designed questionnaire was established by a panel of applied medical sciences faculty at the Jordan University of Science and Technology (JUST). The questionnaire was pilot tested in 10 volunteer parents or caregivers who were asked to complete the questionnaire and provide feedback. Based on the feedback, the questions were modified accordingly. Test-retest reliability was calculated by administering the questionnaire twice to the same individuals (*n* = 10). Cronbach's alpha was used to test the internal reliability of the questionnaire, and the resulting value of 0.75 indicated that the questionnaire items had acceptable internal consistency.

### 2.1. Study Population

The participants with ASD were recruited from special care centers/schools associated with ASD by using a convenience sample from different areas in Jordan. A list of these centers/schools was obtained from the Ministry of Social Development. The centers and the schools were asked to distribute a questionnaire to the parent/caregiver of their members. A paper format of the questionnaire was made available for the parent/caregiver to answer the questions on the respondent's behalf, accompanied by a covering letter explaining the study. The cover letter contained information about the purpose of the research and explained that participation was voluntary, that responses would be anonymous, and provided the principal investigator's contact information.

From August 2018 to February 2019, private and public special care centers associated with ASD in Jordan were visited during normal working hours. Questionnaires were sent to a total of 200 parents/caregivers of individuals with ASD, and 147 of these agreed to participate in our study. The questionnaire included questions addressing participant's demographic information, caregiver information, participant's dental service use, and barriers to access dental care. The questionnaire items relating to barriers to access dental care were obtained and modified from the literature [16–18]. Information regarding the severity of the disability was based on the diagnosis in the patient's record, which was obtained by the principal of the center.

Ten schools and centers agreed to participate in this study. The same questionnaire forms except for a few questions specific to individuals with ASD were sent to 200 individuals without ASD from the same regions, to act as a control group. The control group was selected from the same residences/regions/schools where the cases studied were enrolled.

Our research was conducted in full accordance with the World Medical Association Declaration of Helsinki. The study protocol was approved by the JUST Institutional Review Board (Reference: 2017/0032). The data were anonymized prior to analysis and reported in group form. The return of completed questionnaires was taken as a proxy for consent to participate in the study. The data were analyzed using SPSS Version 22 (IBM Corp., Armonk, NY, USA), with the significance level set at *P* < 0.05. A descriptive analysis of univariate distributions was obtained for each of the 25 questionnaire items. A chi-square test and contingency-table analysis were performed on the data.

Since not all respondents answered each question, the denominator used to calculate the proportions was the total number of nonmissing values.

## 3. Results

From the 200 potential respondents for each group, the response rate was 75% for individuals with ASD and individuals without ASD. The age of the participants varied from 7 to 59 years (mean age of 35 years) in ASD individuals and from 7 to 75 years (mean age of 23 years) in the control group. The male to female ratio in the study population was comparable between the ASD group (71%: 29%) and the control group (67%: 32%). In addition, the average educational level was similar in the two groups (*P* > 0.05), and more than 90% of individuals in both groups were unmarried.  Table 1 demonstrates other demographic items, including the distribution of the ASD centers, the severity of disability among the ASD participants, and the insurance and the family income; there were no significant differences in any of these factors.

There was no statistically significant difference in the length of time since the last visit to a dental service when comparing individuals with ASD and individuals without ASD; the majority last visited the dental clinic less than a year ago (64% and 66%, respectively).

**Figure 1:**
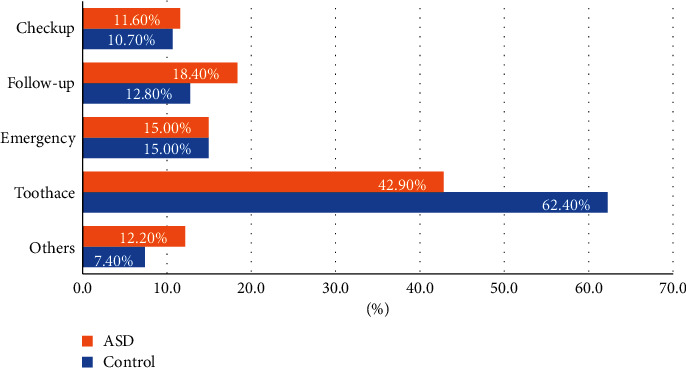
Reasons for last visit to dental service for ASD and control.

Receiving dental treatment for toothache was the main reason for the last visit to dental services in 42.9% of those with ASD and 62.4% of those without ASD, respectively; this difference was statistically significant (*P* < 0.001). Furthermore, 15% of the participants in both groups visited the dental clinic for an emergency. The least frequent reason for the last dental visit given by individuals with ASD was to attend a routine checkup (11.6%; Figure 1).

Table 2 shows the common barriers that prevent individuals from accessing and visiting dental services, analyzed using chi-square testing. There was no significant difference in the likelihood of reporting common barriers such as a long waiting time, high cost, or the absence of any insurance, as well as the inconvenience of dental clinic opening hours, between individuals with ASD and the controls (*P* > 0.05). However, other barriers such as embarrassment (43.5%), a lack of specialized dental staff (28.6%), a lack of knowledge about how to treat people with disabilities (26.6%), and inadequate facilities (34%) were significantly more likely to be reported by individuals with ASD than the controls (*P* < 0.05).

## 4. Discussion

In our study, a similar percentage of participants in the ASD and control groups had visited dentists. Almost two-thirds of ASD participants and control subjects visited the dentist within the preceding year. This was comparable to dentist attendance patterns by ASD children reported in the UK [19] and Saudi Arabia [20]. However, our findings do not agree with a previous study [21] that revealed significant differences between the two groups of participants, as control children visited a dentist significantly more often than children with ASD in Dubai.

The findings of the current study support previous literature showing the difficulties faced by individuals with ASD in accessing professional dental care and locating dental practitioners willing or qualified enough to provide the necessary dental care to this group of individuals [22, 23]. It has been previously reported that the majority of general practitioners are unwilling to treat individuals with ASD [24]. In the current study, about 27% of dentists reported a lack of adequate knowledge to manage people with ASD; this is consistent with a previous parental survey in which dentists' refusal to treat individuals with ASD was attributed to dental education, with 58% of dentists reporting inadequate training to treat children with ASD [23]. This is supported by similar findings where specialized pediatric dentists were reported to treat more individuals with ASD compared to general practitioners, which may be due to specialists receiving more specialized didactic and clinical training in the management of individuals with special needs [25]. General practitioners have previously expressed their willingness to treat individuals with ASD, provided that they received more training in special oral health care needs [24]. The current study findings support the notion that the shortage of specialized dentists such as pediatric dentists is one of the barriers to access appropriate oral health care. A significantly higher number of individuals with ASD cited this as a potential barrier, compared to control participants. Unfortunately, in Jordan, there is no enforced protocol to refer children with ASD from governmental or private clinics to specialist pediatric dentists.

The high rates of uncooperative behavior are an important reason for the unwillingness of dentists to treat individuals with ASD and present an obstacle for these individuals to access appropriate oral health care facilities [26]. Our study demonstrated that fear from dental treatment and feeling of embarrassment were amongst the significant barriers towards seeking dental care. This can be explained by the exaggerated sensory sensitivities of individuals with ASD, lack of psychological or emotional maturity, and their socially averse nature. In a previous study in Saudi Arabia, although a high proportion of children with ASD visited dentists, half of these received only minimal treatment due to their uncooperative behavior [20]. Moreover, cultural attitudes towards ASD, in addition to difficulties in controlling the agitated and anxious behavior of children with ASD, may intensify the feeling of embarrassment among parents. Participants with ASD reported that a nonspacious dental office was a deterrent to receiving dental treatment. The uniqueness of individuals with ASD justifies adopting individualized measures to make the dental office a more comfortable environment for this group of individuals.

In attempting to increase access to oral care by individuals with ASD, one should utilize the least traumatic environment in order to accommodate the unique sensory and behavioral nuances of this population. Multiple and simultaneous strategies, incorporating visual, auditory, as well as tactile modalities and video goggles, could overcome difficulties in communication, social interactions, anxiety, and sensory adaptation among individuals with ASD in the dental office environment [27, 28]. Adaptation strategies in the dental environment have shown to be efficacious in decreasing the negative reactions of individuals with ASD. These measures may reduce the need to resort to more advanced techniques in behavioral management, such as protective stabilization and general anesthesia.

Economic restrictions did not appear to pose a significant barrier to accessing dental treatment in our study. Both the ASD and control groups reported having sufficient finances to cover treatment costs. This is probably because both groups had similar levels of insurance coverage. Most children, and especially children with special needs, are covered by government medical insurance in Jordan. This may also explain, at least in part, the lack of a significant difference between the ASD and control groups in terms of the proportion of individuals that visit a dentist.

The caregivers of individuals with ASD in our study perceived “dental office is too far away” and “inaccessible parking areas” as significant factors influencing their accessibility to dental care. This may reflect an awareness among ASD participants and their families that finding dentists to provide professional specialized care could involve increased travel time and other difficulties [21].

The above discussed barriers to access dental care highlight the crucial need to adopt measures that facilitate access to appropriate oral health care facilities for patients and especially those with ASD. It also reinforces the need to increase the number of dentists qualified in special needs oral health to provide the needed professional dental treatment and education for children with ASD. Our study emphasizes the increased awareness among ASD participants about the need for a qualified dentist. More professional training is required for dentists at the pre- and postdoctoral levels. The corroboration of didactic and clinical material in the undergraduate dental curriculum in Jordan, and fostering graduate training in the dental management of patients with ASD to meet universally acknowledged standards, should be embraced by academic and dental institutions. Furthermore, dentists should be educated to introduce adaptation methods into their practice in order to meet the needs of this group of individuals. Specialized practical guidelines should be disseminated to further educate dentists working with patients with ASD. Children with ASD and their families should also receive appropriate education in special needs oral health care in order to be prepared for dental appointments.

Web-based networks dedicated to the dental care of children with ASD can be valuable in helping families locate dental offices that provide specialized dental treatment to individuals with ASD. These networks can be informative and supportive for this special needs population and increase the awareness and preparedness of children with ASD to receive dental treatment more positively.

There are several limitations to this study. Due to the lack of central autism registry data in Jordan, the current study investigated a group of individuals with ASD identified through their registration at special care centers. This resulted in a selection bias, as those not registered at special care centers and raised at home were not included in this study. In addition, this study was based on information collected by self-reported questionnaires, with answers based on respondents' own subjective interpretation, and this may be biased. The intellectual level of the individuals with ASD was not reported in this study, and knowledge about this could contribute to our understanding of the different obstacles to dental care.

## 5. Conclusions

In our study, a similar number of participants in the control group and ASD group had visited dentists. However, a smaller number of individuals with ASD had attended a dental office for a routine checkup. The most significant barriers towards seeking oral health care perceived by participants with ASD included difficulties in accessing professional dental care, locating dental practitioners adequately qualified to provide care to this group of individuals, nonspacious dental offices or dental offices located too far away, inaccessible parking areas, a fear of dental treatment, and a feeling of embarrassment.

The current study has helped to identify the challenges and barriers associated with dental care for individuals with ASD in Jordan, which could help address and reduce the barriers to dental service utilization among this population. The number of dentists specialized in special needs oral health must be increased to provide dental treatment and education for individuals with ASD. Dentists should be educated to introduce adaptations into their practice in order to meet the needs of this group.

## Figures and Tables

**Table 1:** Sociodemographic characteristics of participants with ASD and control groups.

Characteristics	ASD group		Control group	
	*n*	(%)	*n*	(%)
Family income (JD)				
<250	74	(50.3%)	2	(1.3%)
250–500	58	(39.5%)	70	(47.0%)
500–1000	14	(9.5%)	64	(43.0%)
>1000	1	(0.7%)	13	(8.7%)

Age				
≤18	103	(70%)	97	(65%)
19–40	33	(22.5%)	37	(25%)
>40	11	(7.5%)	15	(10%)

Insurance				
Yes	109	(74.1%)	100	(67.1%)
No	38	(25.9%)	49	(32.9%)

Disability center/region				
Amman	118	(80.3%)	55	(36.9%)
North	25	(17.0%)	68	(45.6%)
South	4	(2.7%)	26	(17.4%)

Severity of disability				
None	0	(0%)	149	(100%)
Mild	41	(27.9%)	0	(0%)
Moderate	65	(44.2%)	0	(0%)
Severe	41	(27.9%)	0	(0%)

**Table 2:** Barriers to dental care among participants with ASD and control groups.

Barrier	ASD, *N* (%)	Control, *N* (%)	*P* value
Could not afford the cost	64 (43.5)	57 (38.3)	0.058
Dental office is too far away	39 (26.5)	19 (12.8)	0.003^*∗*^
Dental office is not open at convenient times	34 (23.1)	40 (26.8)	0.460
Dental office has no or difficult access for wheelchair	61 (41.5)	10 (6.7)	0.001^*∗*^
Dental office has inaccessible parking areas	52 (35.4)	9 (6.0)	0.001^*∗*^
Dental office has a small space	42 (28.6)	7 (4.8)	0.001^*∗*^
Dental office has inadequate facilities to provide dental care	50 (34)	5 (3.4)	0.001^*∗*^
Dentist's lack of knowledge of how to treat people with disability	42 (26.6)	9 (6.1)	0.001^*∗*^
Dental office has a general dentist, not a specialist	42 (28.6)	14 (9.4)	0.001^*∗*^
Long waiting time	72 (49)	62 (41.6)	0.203
Fear of dental work	99 (67.3)	77 (51.7)	0.006^*∗*^
No insurance coverage/dental coverage	57 (39)	52 (34.9)	0.461
Embarrassment or any psychological barriers	64 (43.5)	14 (9.4)	0.001^*∗*^

^*∗*^Significant result, *p* < 0.05.

## Data Availability

BioSample and BioProject code are given. We will notify NCBI for access to genome sequence after publication.
